# The relationship between beliefs in substance craving and quality of life among narcotics anonymous: a cross-sectional study in southeastern Iran

**DOI:** 10.1186/s40359-023-01164-9

**Published:** 2023-04-20

**Authors:** Mahlagha Dehghan, Alireza Malakoutikhah, Homayoon Kazemy, Mohammad Hossein Fattahi Toqroljerdi, Sima Mokhtarabadi, Mohammad Ali Zakeri

**Affiliations:** 1 Department of Critical Care Nursing, Razi Faculty of Nursing and Midwifery, Kerman University of Medical Sciences, Kerman, Iran; 2 M.Sc. in psychiatric nursing, Nursing Research Center, Kerman University of Medical Sciences, Kerman, Iran; 3 Student Research Committee, School of Nursing and Midwifery, Kerman University of Medical Sciences, Kerman, Iran; 4 Student Research Committee, School of Nursing and Midwifery, Kerman University of Medical Sciences, Kerman, Iran; 5 M.Sc. in Medical Surgical Nursing, Shafa Hospital, Kerman University of Medical Sciences, Kerman, Iran; 6 Clinical Research Development Unit, Ali-Ibn Abi-Talib Hospital, Rafsanjan University of Medical Sciences, Rafsanjan, Iran; 7 Non-Communicable Diseases Research Center, Rafsanjan University of Medical Sciences, Rafsanjan, Iran

**Keywords:** Craving beliefs, Substance use, Quality of life, Addiction, Narcotics anonymous

## Abstract

**Background:**

Addiction is a chronic and relapsing disorder characterized by compulsive drug seeking and lasting changes in the brain. Low quality of life may influence the substance craving, which leads to relapsing. Therefore, the present study aimed to investigate the relationship between beliefs in substance craving and quality of life among narcotics anonymous.

**Methods:**

This cross-sectional study was performed on 202 narcotics anonymous in Kerman, southeastern Iran. Convenience sampling was used to select anonymous patients in the Narcotics Anonymous (NA) association in Kerman city. Narcotics anonymous completed the demographic questionnaire, the Craving Beliefs Questionnaire (CBQ), and the World Health Organization Quality of Life-BREF (WHOQOL-BREF). Multiple linear regression model was used to determine the predictors of craving beliefs. The data were then analyzed using SPSS 22.

**Results:**

The mean age of the participants was 38.48 ± 11.32 years. The majority of the samples were male (86.1%), married (65.4%), educated (93.6%) and urban (86.1%). The mean scores of craving beliefs and overall Quality of Life (QOL) were 77.58 ± 20.70 and 64.42 ± 23.13, respectively. Forty-two-point 1% had high level of craving beliefs. We found a significant negative and weak correlation between beliefs in substance craving, physical health domain (r = -0.16, p = 0.02), and overall quality of life (r = -0.15, p = 0.03). History of crack use, job, and physical health domain of QOL were predictors of beliefs in substance craving among the NAs.

**Conclusion:**

Based on the study results, the participants had a high level of substance craving, and some aspects of the quality of life had an impact on the beliefs of addiction. However, it is necessary to conduct more studies in this field; psychological interventions and programs to increase the quality of life may reduce the substance craving.

## Introduction

Addiction is becoming a major problem in different societies and has had many negative effects on the lives of millions of people [1]. Addiction is characterized by the continuous use and compulsive seeking of drugs, and lasting changes in the brain and has raised addiction as a neurological and psychological disorder [2]. People with substance abuse disorder show recurrence of drug-seeking behaviors even after quitting drugs [3]. The rate of relapse in these people after treatment is about 50–90% [4]. Craving is the biggest threat to the recovering patient and the main factor of relapse [5] and the important cause of the addiction relapse after therapeutic interventions [6, 7].

Addiction is associated with compulsive behaviors, disinhibition and sensation seeking, uncontrollable cravings, and continuous consumption. Substance abuse has harmful social, psychological, physical, economic, and familial consequences [8]. Stimuli associated with drug use often have rewarding properties and become conditioned cues that independently cause drug craving [9]. Furthermore, the most important theories of addiction propose craving as the main psychological mechanism in substance use disorders [10].

Narcotics Anonymous or NA founded in 1953 is a global, community-based organization with members of diverse languages and cultures, and provides an ongoing support network for people with addiction who wish to maintain a drug-free lifestyle [11]. NA groups started their activity in Iran in 1990 [12] to improve the self-confidence and provide a warm and supportive environment [13]. According to official reports in Iran, more than 1.12 million people aged 15–64 years old have drug use disorders (2.1%). Opioids are the most common type of drug use disorders in Iran [14].

Drug addiction has gained a global scope, gone beyond the boundaries of health and treatment, and turned into a socio-familial problem [15]. Substance abuse is one of the primary mental health problems in the Iranian population [14]. Studies suggests that drug abuse reduces the quality of life [16, 17]. The quality of life includes mental and physical aspects. Negative physical aspects are characterized by physical weakness, while negative mental aspects include depression, anxiety, and the breakdown of family relationships [18]. Studies have shown a correlation between reduced quality of life in people with addiction and the physical and psychological consequences of addiction [19], and various researchers and theorists considered low quality of life as the most important cause of substance abuse relapse [20].

NA are from all strata of society and limited studies addressed health and quality of life of these people. The substance craving is an important factor in the recurrence of drug use and many factors affect it. It seems necessary to conduct more studies on the quality of life and the substance craving to prevent the return to drug use and improve the quality of life. Therefore, this study aimed to investigate the relationship between beliefs in substance craving and quality of life among the NAs.

## Method and materials

### Study design and setting

This cross-sectional study was conducted on the relationship between beliefs in substance craving and quality of life among the NAs in Iran. Data were collected from April to September 2021.

### Sampling and sample size

After obtaining the necessary permits, the researcher referred to the Narcotics Anonymous Association in Kerman. The study population was all men and women participating in the NA meetings. The inclusion criteria were educated participants aged above 18 years old, who were diagnosed with substance dependence. Incomplete questionnaires were excluded from the study. To estimate the sample size, we used Cochran’s formula for infinite population (Z = 1.96, d = 0.07, n = 196). Based on the dropout rate, 240 questionnaires were distributed. Finally, 214 participants completed the questionnaires, and the response rate was 89.16%. After removing the incomplete questionnaires, the data of 202 participants were used in the final analysis.

### Measures

#### Demographic characteristics information form

The demographic characteristics information form included age, sex, marital status, education level, job, living place, age of starting drug use, history of drug use, history of secession, Being an NA member, history of mental disorders, history of physical disorders, history of opium usage, history of different drug use, history of family member addiction, and history of unsuccessful secession.

#### Craving beliefs Questionnaire (CBQ)

The 20-item self-report CBQ measures beliefs in substance craving. Each question was graded on a 7-point Likert scale (from completely agree = 7 to completely disagree = 1). To obtain the total score, the scores obtained from each question were added with each other. The minimum and maximum scores were 20 and 140, respectively. Scores 20–50, 50–80, and above 80 indicated low, moderate, and high levels of craving beliefs, respectively [21]. Küçükkarap et al. (2018) investigated the validity and reliability of CBQ, and its Cronbach’s alpha coefficient was 0.94 [22]. Mohammadzadeh (2018) reported Cronbach’s alpha coefficient of 0.84 in Iran [23].

#### The World Health Organization Quality of Life (WHOQOL)-BREF

The 26-item WHOQOL-BREF measures the quality of life in four areas of physical health, mental health, social relations, and environmental health. In this questionnaire, the first two questions do not belong to any of the areas and examine the health status and overall quality of life. It is necessary to grade questions 3, 4, and 26 inversely. The minimum and maximum scores of each area were 4 and 20, which must be converted into a score of 0-100. Nejat et al. (2005) investigated the questionnaire validity and reliability in Iran, and its reliability using Cronbach’s alpha coefficient was 0.77, 0.77, 0.55, and 0.84 for the physical health, the mental health, the social relations, and the environmental health, respectively [24].

### Data collection

After obtaining the necessary permits, the researcher invited all eligible individuals participated in the NA association to complete the questionnaire when they were ready.

### Data analysis

Descriptive and inferential statistics, as well as SPSS 25 were used to analyze the data. Descriptive statistics were used to describe demographic characteristics and mean scores. The Pearson correlation coefficient was used to determine the relationship between the beliefs in substance craving and the QOL, while the Mann-Whitney U test, independent t-test, and analysis of variance were used to determine the relationship between the beliefs in substance craving and demographic and clinical characteristics. Multiple linear regression model was used to determine the predictors of craving beliefs about substance use. All variables with p value < 0.2 in bivariate analysis included in the multiple linear regression model (job, history of crack use, history of using different type of substances, history of family member addiction, physical health domain and overall quality of life). A significance level of 0.05 was considered.

## Results

### Participants’ demographic and clinical characteristics

The mean age of the participants was 38.48 ± 11.32 years (Min = 19 and Max = 76). The majority of the samples were male (n = 174; 86.1%), married (n = 132; 65.4%), educated (n = 189; 93.6%), employed (n = 157; 77.7%), and urban (n = 174; 86.1%) (Table 1). The majority of the participants had started using substance when they were 19–30 years old for more than five years. The majority of the participants had no history of mental or physical disorders. The majority had history of opium use (Table 2).

**Table 1:** Demographic characteristics of the participants and their relation with craving beliefs about substance use among the participants

Variable	Frequency (percent)	Craving beliefs about substance use		Statistical test (P value)
		Mean	SD	
Age (yr.)				
≤ 30	55 (27.2)	79.0	17.98	F = 0.22 (0.89)
31–40	75 (37.1)	77.11	21.28	
41–50	46 (22.8)	76.0	23.58	
> 50	26 (12.9)	78.77	19.82	
Sex				
Female	28 (13.9)	73.04	24.53	t = -1.26 (0.21)
Male	174 (86.1)	78.32	19.99	
Marital status				
Single	56 (27.7)	76.67	20.57	F = 0.42 (0.66)
Married	132 (65.4)	78.93	19.30	
Divorced/widow(er)	14 (6.9)	80.86	17.46	
Education level				
Illiterate	13 (6.4)	78.15	10.25	F = 0.65 (0.58)
< Diploma	84 (41.6)	79.06	22.80	
Diploma	60 (29.7)	78.20	22.18	
Academic	45 (22.3)	73.84	16.38	
Job				
Employed	157 (77.7)	75.33	19.74	t = -2.94 (0.004)
Unemployed	45 (22.3)	85.44	22.21	
Living place				
Urban	174 (86.1)	77.15	22.19	Z = -1.07 (0.28)
Rural	28 (13.9)	80.29	4.99	

SD = Standard Deviation, t = Independent t test, F = Analysis of variance, Z = Mann-Whitney U

**Table 2:** Clinical characteristics of the participants and their relation with craving beliefs about substance use

Variable	Frequency (percent)	Craving beliefs about substance use		Statistical test (P value)
		Mean	SD	
Age of starting drug use (yr.)				
≤ 18	69 (34.2)	80.07	21.35	F = 0.98 (0.38)
19–30	120 (59.4)	76.69	20.57	
> 30	13 (6.4)	72.62	17.99	
History of drug use (yr.)				
< 5	49 (24.3)	77.78	18.01	F = 0.01 (0.99)
5–10	68 (33.7)	77.81	18.62	
> 10	85 (42.0)	77.29	23.72	
History of secession (yr.)				
< 5	130 (64.4)	77.13	19.94	F = 0.42 (0.66)
5–10	53 (26.2)	79.60	19.34	
> 10	19 (9.4)	75.05	28.84	
Being an NA member (yr.)				
< 5	122 (60.4)	77.25	19.44	F = 1.07 (0.34)
5–10	55 (27.2)	80.33	20.88	
> 10	25 (12.4)	73.16	25.76	
History of mental disorders				
Yes	25 (12.4)	83.32	25.59	t = 1.48 (0.14)
No	177 (87.6)	76.77	19.86	
History of physical disorders				
Yes	28 (13.9)	76.11	26.16	Z = -0.08 (0.94)
No	174 (86.1)	77.82	19.76	
History of opium use				
Yes	148 (73.30	76.71	19.13	Z = -0.76 (0.45)
No	54 (26.7)	79.98	24.53	
History of Shisheh use				
Yes	78 (38.6)	79.64	20.41	t = -1.12 (0.26)
No	124 (61.4)	76.29	20.85	
History of crack use				
Yes	20 (9.9)	89.95	17.94	t = -2.86 (0.005)
No	182 (90.1)	76.22	20.57	
History of marijuana use				
Yes	33 (16.3)	78.91	18.49	t = -0.40 (0.69)
No	169 (83.7)	77.32	21.14	
History of cocaine use				
Yes	11 (5.4)	77.45	22.88	t = 0.02 (0.98)
No	191 (94.6)	77.59	20.63	
History of other drug use				
Yes	53 (26.2)	75.57	25.06	Z = -0.04 (0.97)
No	149 (73.8)	78.30	18.95	
History of using different type of substances				
One type	105 (52.0)	77.62	20.58	F = 2.52 (0.06)
Two types	64 (31.7)	78.30	21.59	
Three types	25 (12.3)	70.64	18.49	
≥ Four types	8 (4.0)	93.12	13.42	
History of family member addiction				
Yes	164 (81.2)	78.68	19.33	Z = -1.78 (0.07)
No	38 (18.8)	72.84	25.54	
History of unsuccessful secession				
Yes	167 (82.7)	78.38	19.46	t = 1.2 (0.23)
No	35 (17.3)	73.77	25.80	

SD = Standard Deviation, NA = Narcotics Anonymous t = Independent t test, F = Analysis of variance, Z = Mann-Whitney U

### Beliefs in substance craving

The mean score of beliefs in substance craving was 77.58 ± 20.70 (Table 3). Nine-point 9% (n = 20) of the participants had low level of beliefs in substance craving, 48.0% (n = 97) had moderate level of beliefs in substance craving, and 42.1% (n = 85) had high level of beliefs in substance craving.

**Table 3:** **The craving beliefs about substance use and quality of life and their association among the participants**

Variable	Mean	SD	Minimum	Maximum	Craving beliefs about substance use	
					r	P value
Craving beliefs about substance use	77.58	20.70	22	139	-	-
Physical health domain	60.43	18.0	3.57	100	-0.16	0.02
Psychological health domain	56.08	13.77	8.33	100	0.07	0.30
Social relationships domain	61.34	21.57	0.0	100	-0.05	0.47
Environment health domain	53.76	15.18	12.50	96.88	0.04	0.54
Overall quality of life	64.42	23.13	0.0	100	-0.15	0.03

SD: Standard deviation, r = Pearson Correlation Coefficient

### Quality of life among narcotics anonymous

The mean scores of physical health, psychological health domains of the WHOQOL-BREF, social relationships, environmental health, and the QOL and general health were 60.43 ± 18.0, 56.08 ± 13.77, 61.34 ± 21.57, 53.76 ± 15.18, and 64.42 ± 23.13, respectively. Therefore, the highest score belonged to the overall QOL and general health, while the lowest score belonged to the environmental health domain. The mean scores of all domains of the QOL were above the scale midpoint of 50.

### The association between beliefs in substance craving, quality of life, and other study variables among narcotics anonymous

The present study indicated a significant negative and weak correlation between beliefs in craving substance, physical health domain (r = -0.16, p = 0.02), and overall quality of life (r = -0.15, p = 0.03) but no correlation between beliefs in craving substance and other domains of the QOL (Table 3). Among other study variables, employed participants had significantly lower level of beliefs in craving substance than unemployed participants. In addition, participants with history of crack use had higher level of beliefs in substance craving than others (Tables 1 and 2). For further analysis, we used multiple linear regression and considered beliefs in craving substance as dependent variable and history of crack use (Yes/no), job (employed/unemployed), physical health domain of QOL, and overall quality of life as independent variables. The results showed that history of crack use (p = 0.006), job (p = 0.01), and physical health domain of the QOL (p = 0.046) were predictors of beliefs in craving substance among the NAs (Table 4).

**Table 4:** Multiple regression analysis summary for craving beliefs about substance use

Variable	Unstandardized Coefficients		Standardaized Coefficients	t	P value	95% Confidence for B	
	B	Standard error	Beta			Lower Bound	Upper Bound
Constant	92.45	5.3		17.44	< 0.001	82.0	102.90
Job	-8.51	3.40	-0.17	-2.50	0.01	-15.21	-1.80
History of crack use	12.89	4.68	0.19	2.75	0.006	3.66	22.12
Physical health domain of QOL	-0.16	0.08	-0.14	-2.0	0.046	-0.31	-0.003

QOL: Quality of life, Adjusted R^2^ = 0.08, F = 6.90, P < 0.001; Job (Unemployed = 0 and Unemployed = 1); History of crack use (No = 0 and Yes = 1)

## Discussion

The present study aimed to investigate the relationship between beliefs in substance craving and quality of life among the NAs and found that the participants had a high level of substance craving, and some aspects of the quality of life had an impact on the beliefs of addiction.

In the present study, 42.1% had a high level of beliefs in craving. The review of the literature showed that similar studies did not address the percentage of beliefs in substance craving. Other studies reported moderate level of beliefs in substance craving due to the variety of drug types. Yaghubi et al. indicated that the mean beliefs in methadone craving in the control and intervention groups were 83.37 ± 17.54 and 89.52 ± 14.34, respectively before the intervention [25], which were more than that in the present study. Ahmadpanah et al. reported that the mean score of methadone craving in the control and intervention groups were 49.21 ± 15.19 and 51.00 ± 15.78, respectively before the intervention [26]. Patients’ alcohol craving was 68,10 ± 30,30 [27], which was lower than the average level of beliefs in craving in the present study (77.58 ± 20.70). These findings showed that increased cravings in drug-dependent patients might be one of the factors that made them more vulnerable to opioid addiction.

Hugh et al. in the United States supported our study and showed that each 1-unit increase in the craving scale was associated with a 31% higher likelihood of drinking in the next week [7]. Stohs et al. in the United States found a significant relationship between the alcohol relapse after treatment and increased craving [28]. The results of the aforementioned studies clearly explained the importance of craving in the relapse of drug use, which could affect the quality of life of people with addiction.

The study results showed a negative and weak correlation between the beliefs in substance craving, physical health and overall quality of life, so we must interpret the results with caution and conduct more studies in this regard. The present study indicated that the mean scores of all areas of quality of life were higher than the midpoint of the scale, but similar studies reported the average mean scores were lower than that in the present study. Barati et al. showed a low quality of life among drug dependent women [17] and Muller et al. reported a poor quality of life among people with drug addiction [29]. Beliefs in substance craving leads to irritability, aggression, and other psychological symptoms, reduces physical performance, weakens psychosocial capabilities, and reduces people’s quality of life [30]. The quality of life depends on several factors such as the mental health, which can be affected by the beliefs in substance [31]. Jenkins et al. in the United States showed a strong relationship between negative emotions and cravings [32]. Giménez-Meseguer et al. indicated a significant reduction in substance use among people with a higher quality of life [16]. The results of the aforementioned studies are consistent with the results of the present study, but we need more studies in this regard due to the poor relationship between quality of life and craving beliefs in the current study. According to a review of the literature, various studies suggested that improved quality of life could effectively reduce substance uses during treatment [33, 34]. Therefore, it is necessary to design psychological interventions to improve mental health, quality of life and reduce craving among addicted people.

The study results showed that history of crack use, occupation, and physical health domain of quality of life were predictors of beliefs in substance craving among the NAs. The review of the literature suggested no similar study in this regard. Studies pointed out the effects of other variables on cravings. Fatseas et al. showed that anxiety and/or mood disorders were associated with higher intensity of craving and frequent substance use [35]. Mason et al. found a significant relationship between spirituality, self-efficacy, and craving [36]. Zhang et al. showed that physical activity had a positive effect on reducing the desire for drugs in people with substance-use disorders [37]. Delonca et al. confirmed the significant effects of exposure to alcohol images and metacognitions on craving [38]. As we found no similar study regarding craving predictors, it is necessary to pay attention to these results in future studies.

Previous studies demonstrated a relationship between the level of craving responses, intra-individual and inter-individual variables [39]. The variety of opioids and the ways people cope with quitting these substances are other things that can affect cravings. Fatseas et al. showed that patients who used opioid, tobacco, and alcohol were more likely to receive pharmaceutical treatments for addiction [35], which might have affected the quality of life of these people and reduced the substance craving. The self-report measure of craving does not evaluate multidimensional characteristics; therefore, some authors have suggested the existence of different types of craving [40]. We need further studies to address the mechanisms through which various factors such as quality of life can affect consumption and craving intensity.

### Limitations

This study had several limitations. The current study was cross-sectional and did not investigate the causal relationship. We need further studies in this regard due to the few similar studies in the literature review. The use of self-reporting tools to assess cravings is also one of the limitations of the present study. Therefore, it is suggested to use more accurate measurement methods such as psychiatric interview. This study was conducted in one of the cities in southeastern Iran, so it is necessary to conduct this research in other areas and different cultures in order to generalize the results.

## Conclusion

The study results reported a high level of drug addiction and a weak relationship between physical health, general quality of life, and craving beliefs, so it is necessary to pay attention to the level of the quality of life in people with addiction. According to the results, it is necessary to conduct more studies in this field. Some variables such as history of crack use, job, and physical health were influential on the craving beliefs, but it is necessary to conduct more studies in this regard, as well as to carry out psychological interventions to increase the quality of life and reduce the cravings. The study results provide a new insight into the perception of the variables influencing on drug addiction in order to plan and manage this problem.

## Data Availability

The datasets used and/or analysed during the current study available from the corresponding author on reasonable request.
